# Improving the Quality of Consumer Health Information on Wikipedia: Case Series

**DOI:** 10.2196/12450

**Published:** 2019-03-18

**Authors:** Shira Schecter Weiner, Jill Horbacewicz, Lane Rasberry, Yocheved Bensinger-Brody

**Affiliations:** 1 Doctor of Physical Therapy Program School of Health Science Touro College New York, NY United States; 2 Data Science Institute University of Virginia Charlottesville, VA United States

**Keywords:** consumer health information, health literacy, Wikipedia, public health, physical therapy education

## Abstract

**Background:**

Wikipedia is one of the most consulted health resources in the world. Since the public is using health information from Wikipedia to make health care decisions, improving the quality of that health information is in the public interest. The open editable content design of Wikipedia and quality control processes in place provide an opportunity to add high-value, evidence-based information and take an active role in improving the health care information infrastructure.

**Objective:**

The aim of this project was to enhance Wikipedia health pages using high-quality, current research findings and track the persistence of those edits and number of page views after the changes to assess the reach of this initiative.

**Methods:**

We conducted Wikipedia Editathons with 3 different cohorts of Physical Therapy (PT) students to add high-quality health information to existing Wikipedia pages. Students synthesized best evidence information and updated and/or corrected existing Wikipedia entries on specific health pages. To evaluate the impact of these contributions, we examined two factors: (1) response to our contributions from the Wikipedia editing community, including number and type of subsequent edits as well as persistence of the student contributions and (2) number of page views by the public from the time of the page edits.

**Results:**

A total of 98 PT students in 3 different cohorts engaged in Editathons, editing 24 health pages. Of the 24 edits, 22 persisted at the end of the observation period (from time of entry to May 31, 2018) and received nearly 8 million page views. Each health page had an average of 354,724 page views.

**Conclusions:**

The Wikipedia Editathon is an effective way to continuously enhance the quality of health information available on Wikipedia. It is also an excellent way of bridging health technology with best-evidence medical facts and disseminating accurate, useful information to the public.

## Introduction

Wikipedia is one of the most, if not the most, consulted health resources in the world [1]. Therefore, improving the quality of its content ensures the dissemination of high-level information to all who visit its pages. The open editable content design of Wikipedia and the quality control processes in place provide an opportunity to add high-value, evidence-based information and take an active role in improving the health care information infrastructure.

The internet is a major source for the dissemination of health information. Research has shown that 50% of American adults seek health information from online resources [2]. This information can potentially influence health beliefs and health behaviors. The US government set the following goal in its Healthy People 2020 initiative: “use health communication strategies and health information technology to improve population health outcomes and health care quality, and to achieve health equity.” The internet’s reach and potential for improving the health of individuals as well as populations should be recognized by health care professionals as another tool in their toolbox to improve health care quality and equity.

When accessing information on the internet, users begin with a search engine and then select from the results. Wikipedia, which launched in 2001 as a free online encyclopedia, is frequently in one of the top five search result options. Traffic to Wikipedia’s English-language medical content places it in the lead among other health internet sites, including WebMD and Mayo Clinic [1]. The English-language Wikipedia held approximately 30,000 articles on health-related topics in 2017 [3]. Across Wikipedia encyclopedias, in all languages, there were 155,000 health articles, which collectively received 4.8 billion page views in 2013. Those articles contained 950,000 citations [1]. The page view counts continue to increase with monthly averages estimated at 170 million views between July 2009 and April 2018, approximating more than 16 billion views during this period (see Multimedia Appendix 1 for spreadsheet data on page views).

Despite the fact that Wikipedia is a primary source for public health information, many health care professionals reject Wikipedia as a reliable source of information because of the open editable content. A more detailed understanding of Wikipedia’s inner workings highlights the quality control processes in place to ensure inclusion of worthwhile information and detect misinformation. Because the articles are wiki documents, they are continuously editable and never considered final. Volunteer editors from all over the globe edit Wikipedia entries, and a history of all changes is tracked within the page and available for review. This nontraditional editorial model allows anyone with an internet connection to contribute and make changes. Typically, subject-matter experts, such as health care professionals and medical students, take on this role [4]. Therefore, as evidence-supported information from reliable and verifiable sources is added and misinformation or outdated content is removed, the quality of the information presented is continually enhanced. This is something that cannot be said for traditional publications. Using this crowdsourcing model facilitates disseminating a great deal of high-level information to large segments of the population.

Researchers have begun delving deeper into the quality of Wikipedia health content, utility of citations, and the editorial process. The Wikipedia standards demand the inclusion of citations, while most internet health sources do not impose this same standard. Hunter et al [5] compared both content and the relevant use of supporting references on Lexicomp (an online drug information source for clinicians) and Wikipedia. The results showed that although Wikipedia contained less content overall, all Wikipedia content was supported by peer-reviewed citations, while the same was true for only 63% of the Lexicomp information [5]. It has also been noted that the citations in Wikipedia’s health pages become a “gateway to biomedical research,” as clinicians use these links to launch further investigation into a particular topic [6].

A report by the Intercontinental Marketing Services Institute for Healthcare Informatics revealed that as many as 96% of all edits made in Wikipedia health pages are geared toward the patient [7]. It stands to reason that if the public uses the health information from Wikipedia to make health care decisions, improving the quality of that health information is in the public interest. While much has been written about the open editable content, the impact of the edits has not been assessed in terms of their reach. The purpose of editing the pages is to improve the health information retrieved by the public. Therefore, examining the page views since the edits is a necessary part of that process.

The aim of this project was to enhance Wikipedia health pages using high-quality, current research findings and track the persistence of those edits and the number of page views after the changes. Page views are reflective of public interest in a topic, with more page views demonstrating greater public interest. The Touro College Doctor of Physical Therapy (PT) program initiated Wikipedia Editathons supported by a grant from Consumer Reports magazine to add high-quality health information to existing Wikipedia pages. By adding accurate, current, best evidence to specific Wikipedia pages in layman’s terms, enhanced information can be widely disseminated. Consumers benefit by using information that can guide evidence-based health decisions. Clinicians benefit by engaging with patients who are well informed, prepared to partner in their health care process and aware of self-care strategies that may promote well-being. It is important to note that increasing page readership was not a goal of this initiative, as attracting readers to specific pages was beyond the scope of this project.

## Methods

The Editathon is a 3-hour event that is embedded within an existing course in the PT curriculum. The goal was for each student to add, at minimum, one claim backed with one citation to one Wikipedia page.

### Preparing for the Editathon

The preparation for the Editathon involved several steps prior to the day of the actual event. First, we educated the students on how to search the literature and how to assess quality evidence. All students received 6 hours of training from two experienced physical therapy researchers on where to search, how to search, and how to assess the quality of sources within their physical therapy coursework. The next step was for each student to create a personal Wikipedia account and username. Simultaneously, we set up a program page under the “programs and events” dashboard in Wikipedia (meta.wikimedia.org/ wiki/Programs_&_Events_Dashboard) listing all of the editors in the cohort.

Working closely with a resident Wikipedian (Wikipedia expert), we then used Wikipedia’s tracking and algorithms to identify the pages with the greatest need for updates, defined as pages that were frequently visited, with high page views, but with a low number of supporting citations. The focus on frequently visited pages suggests that these topics are of great interest to the public, and the lack of citations demonstrates the need for editorial update. While the number of citations tracked by Wikipedia does not infer quality of those citations on those health pages, our directive was to add high-quality findings from recent (within 5 years when possible) systematic reviews. Those health pages that could be enhanced by physical therapy information were specifically identified for inclusion and added to the program’s event dashboard. It should be noted that some topics that are listed on our dashboard are closely related. This is because some common conditions are referred to by different terms and therefore have distinct Wikipedia pages. One such example is back pain and low back pain. The presence of both pages may foster greater access to the public based on preferred user terminology.

We established small working student groups and each group chose a topic of interest. Groups began by reading the chosen Wikipedia page looking for any PT-related information or lack thereof as well as the accuracy of the information and the recency and quality of the citations. While reading the chosen page, students asked themselves the following as they relate to PT: (1) What would I have liked to know more about when reading the Wikipedia entry? (2) What more might others who seek out this Wikipedia page like to know about this topic? (3) Is there more current evidence that should be added or more recent references that might better support the topic? We instructed students to locate 3 to 5 systematic reviews (Cochrane Reviews when available) that were less than 5 years from publication and related to the topic of interest. Of primary importance was that the reference could provide information that may complement, update, or correct the existing Wikipedia entry and better inform the reader of this topic. The preparatory process concluded with the reading, synthesis, and extraction of information to be added to the Wikipedia pages. Edits were reviewed by faculty and finalized prior to the Editathon.

### Executing the Editathon

During the first half of the Editathon, a Wikipedia expert educated students on the scope, infrastructure, and metrics maintained by Wikipedia. The students were instructed on the editing process for adding information to a Wikipedia page and adding citations for these edits. The goal of the day was for each student to contribute a minimum of one edit ranging from a sentence to a paragraph along with corresponding references. The Wikipedian and faculty agreed that no deletions were to be made of existing Wikipedia entries during this Editathon. If students identified information on a page that contradicted current evidence, they were advised to use a format that recognized the previous information while providing updated accurate information along with supporting citations (for example, “While previous conventional wisdom suggested [erroneous information], more recent evidence suggests [current, accurate information]”). During the second half of the Editathon, students entered their edits and citations on their pages. As this process was finalized, students monitored their entries for responses in real time from the Wikipedia community at large by monitoring the edit link of their topic. At the conclusion of the Editathon, the facilitator shared suggestions for participants to follow-up on their changes, such as returning to Wikipedia to receive feedback on their contributions from editors-at-large or view audience traffic for the edited articles. The facilitator also encouraged all workshop participants to continue to use their account and edit live Wikipedia articles independent of the Editathon.

### Analysis

To evaluate the impact of our contributions on the dissemination of health information, we examined two specific factors: number of page views by the public since the time of the page edits and response to our contributions from the Wikipedia editing community including number and type of subsequent edits and persistence of the student contributions. Page views prior to the Editathon were used to identify pages with considerable traffic but were not considered in the outcome, as the primary purpose was to monitor the rate of dissemination from the time of the student contributions. Page views were recorded for 4 months for all pages following each Editathon. Page views were only tracked if the edit persisted. In addition, we monitored total page views from the inception of each of the 3 cohort Editathons through May 31, 2018. While time frames vary for follow-up as the Editathons took place in 3 different years, total views are relevant since our target was health information dissemination.

As no standard methodology exists to describe the impact of a new Wikipedia contribution, we developed our own systematic approach, which is easily reproducible. We ran the Editathon in the same 1-day format annually for 3 years. Our observation period began on the day the students submitted their edits, March 29, 2016, April 4, 2017, or February 5, 2018, and ended on May 31, 2018.

Using the history tab on each edited Wikipedia page, we identified the log of the student edits by student username and timestamp of the edit. We first insured that only those contributions that met the criteria of adding new health-related content with an acceptable citation to an academic source were included in our metrics. We then used the “compare selected revisions” link (also known as “track changes”) located within the history tab to compare the text of a Wikipedia article as it existed at 2 different points in time—at the time it was added and on May 31, 2018, our end date.

All edits that we tracked were categorized as those that persisted (contribution remained on the page in original or modified format) or those that did not (contribution removed). For all edits that persisted, we further subdivided the responses into 3 categories based on how subsequent Wikipedia editors altered the content: (1) additions (student information was enhanced), (2) partial deletions, or (3) copy edits. We also tracked the number of subsequent edits and the number of editors who made those edits. To assess our impact on dissemination of health information for all edits that persisted, we used the “page view statistics” link to access the count of Web traffic on each Wikipedia page. We began our page view counts from the date of the Editathon through a 4-month follow-up period and monitored page counts for all Editathons collectively through May 31, 2018, the end of the observation period.

## Results

As of the end of the observation period, 98 PT students in 3 different cohorts engaged in Editathons. Students edited 26 Wikipedia articles, with 7 articles in the March 2016 cohort, 8 articles in the April 2017 cohort, and 11 articles in the February 2018 cohort. Of these 26 articles, 24 articles met the criteria of having a student add health information and a relevant citation. The other 2 articles involved student copy editing without making a claim or adding a citation. Of the 24 edits, 22 persisted at the end of the observation period.

Wikipedia’s in-platform communication system solicits a diverse base of editors to check recent editorial changes. From the date of edit through May 31, 2018, the 24 articles received between 2 and 556 edits each. Edits include those made in direct response to the student additions and other changes to the content between the time of student entries through the completion of the observation period. On average, each article received 90 subsequent edits, with a median of 36 edits per target article. While time frames vary for follow-up, as the Editathons took place in 3 different years, our target was sustainability of student entries and thus total edits are relevant. Subsequent edits may be influenced by newly published or conflicting research findings. In 17 of the 24 cases, the Wikipedia community editorial response was entered within 1 day, which is typical for new content submissions. This demonstrates the attentiveness of the Wikipedia editors at large, who monitor for changes and quality control. For the remaining 7 articles, the response time ranged from 11 to 323 days, with a median response time of 23 days. The 24 articles had 16 different reviewing editors inserting a wide range of edits (Table 1).

**Figure 1:**
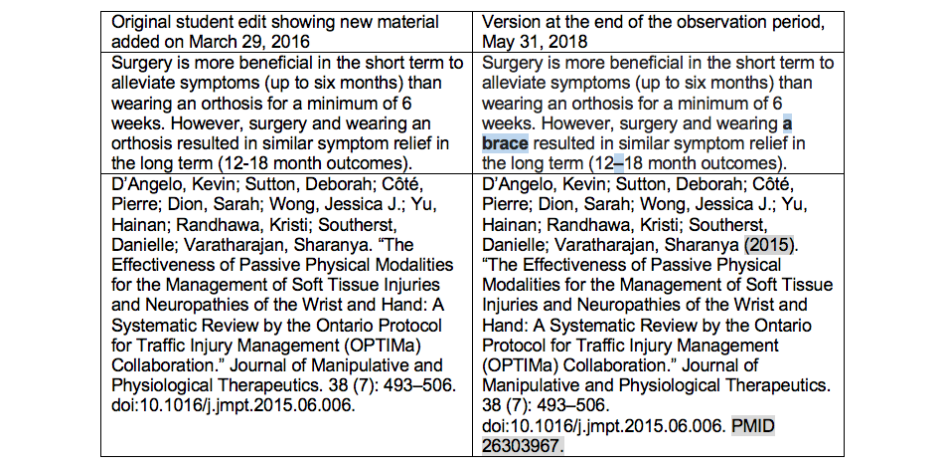
Edits to the Wikipedia page titled Carpal Tunnel Syndrome at the editing event on March 29, 2016.

Two examples that illustrate the outcome of the editorial process are highlighted. In the first example, a student added 2 sentences related to the evidence regarding surgery and bracing for carpal tunnel syndrome to the Carpal Tunnel Wikipedia page and cited a systematic review from 2015 during the March 29, 2016, Editathon (Figure 1). The original contribution is on the left and the version of that page as of May 31, 2018, is on the right.

In this example, a Wikipedia community reviewer changed the student’s use of “an orthosis” to “a brace” to meet Wikipedia's Manual of Style for Medicine, which targets a lay audience. Users operating semiautomated Wikipedia copyediting tools made subsequent edits to add the date of publication and a PubMed identifier (PMID) to the citation and convert a hyphen to an en dash. For our statistics on this contribution, we judged that the student edits persisted and noted all of the changes described above as copy edits. There were no sentence or citation deletions noted for this entry.

**Table 1:** Editorial activity on Wikipedia within 24 hours of student contributions.

Distinct Wikipedia editors	Instances when editor responded within 24 hours
Editor 1	10
Editor 2	5
Editor 3	2
Editor 4	2
Editors 5-16	12 (1 per editor)

**Figure 2:**
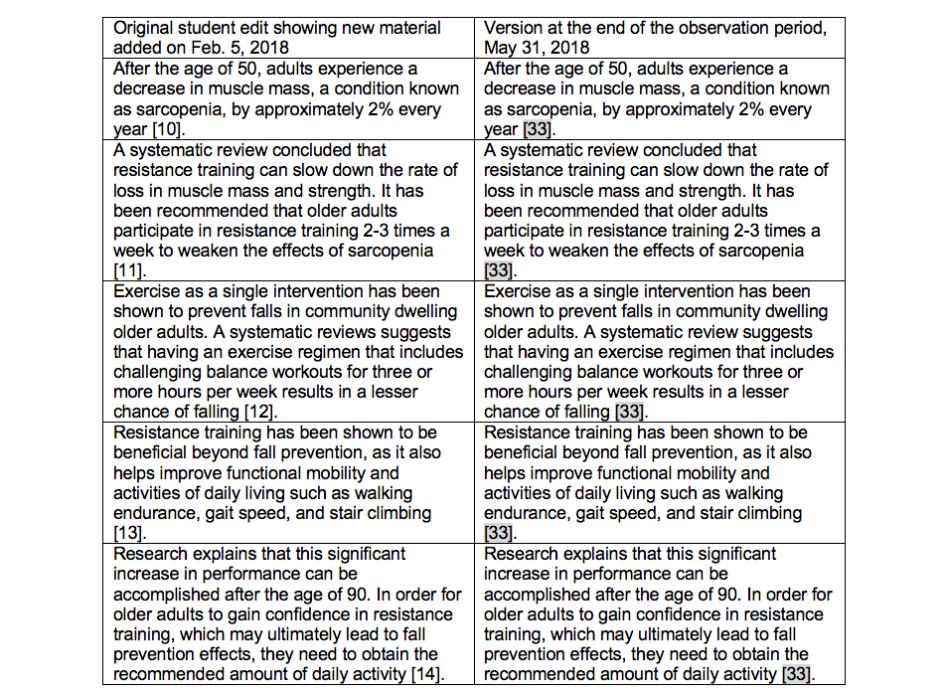
Edits to the Wikipedia page titled Fall Prevention at the editing event on February 5, 2018.

The second example (see Figure 2) describes an edit added to the Fall Prevention health page from the Editathon held on February 5, 2018. The student summarized information from a 2017 systematic review demonstrating that resistance training leads to enhanced functional mobility such as improvement in balance and reduction in falls. The student contributed to the page with 5 separate additions of new information that would be useful to someone seeking information on how to prevent falls and added the systematic review as the reference for each edit. The next day, a Wikipedia editor noted that the systematic review was listed as a new source each time and posted as citations 10, 11, 12, 13, and 14. The editor combined the repeated citations but did not make any changes to the actual content of the edits. At the most recent review of the page on May 31, 2018, all the edits persisted and the number of the citations, originally 10, was now 33, indicating that 23 more references had been added to the page above the section edited by the student.

For our statistics on this contribution, we judged this contribution as follows: the student edits persisted and the contribution underwent copy edits. The total number of edits by the Wikipedia community to the 22 persisting contributions are described in Table 2.

**Table 2:** Summary of edits to 22 Wikipedia pages with persisting contributions.

Change type	Amount (n)	Description of change
Additions	10	Enhanced student prose or replaced a student citation with an alternative relevant citation
Partial deletions	6	Deleted at least one sentence of prose
	3	Shortened student explanation
	5	Kept at least one citation and deleted at least one citation
Copy edits	21	Changes to software code, punctuation, or citation format

**Table 3:** Page views from time of contribution through May 31, 2018, and within 4 months after the edit (for years 2016 and 2017).

PT^a^-relevant Wikipedia page	Date of initial edit	Page views through May 31, 2018	Page views at 4 months from entry
Back pain	March 29, 2016	700,575	98,613
Carpal tunnel syndrome	March 29, 2016	2,374,443	357,717
Developmental coordination disorder	March 29, 2016	684,635	120,323
Patient education	March 29, 2016	43,405	5871
Physical exercise	March 29, 2016	167,147	175,413
Urinary incontinence	March 29, 2016	774,051	102,126
Sports injury	March 29, 2016	150,767	19,645
Sports injury^b^	April 4, 2017	—	19,903
Chronic pain	April 4, 2017	165,432	49,267
Joint dislocation	April 4, 2017	89,410	23,080
Physical therapy	April 4, 2017	666,181	191,090
Physical therapy education	April 4, 2017	51,875	17,894
Spinal cord injury	April 4, 2017	307,377	83,308
Spinal disease	April 4, 2017	37,006	9453
Telerehabilitation	April 4, 2017	10,182	2849
Angina	February 5, 2018	218,465	218,465
Cerebral palsy	February 5, 2018	539,542	539,542
Chronic obstructive pulmonary disease	February 5, 2018	484,912	484,912
Disabled sports	February 5, 2018	19,324	19,324
Fall prevention	February 5, 2018	5135	5135
Low back pain	February 5, 2018	162,590	162,590
Plagiocephaly	February 5, 2018	50,988	50,988
Prosthesis	February 5, 2018	100,478	100,478

^a^PT: physical therapy.

^b^The same topic was edited during two Editathons—page views represents sum total from time of initial edit.

Last, to measure impact using the Wikipedia Pageviews Analysis tool, we found that the 22 articles with persisting student edits received 7,803,920 page views (Table 3). The number of page views from date of the student entries through the first 4 months after the entries and through May 31, 2018, are shown. Four months was chosen to compare activity on each edited page for the same time frame across the different years. At the end of the observation period, each edited Wikipedia health page had an average of 354,724 page views while the median number of page views was 164,011. It is interesting to note the student-edited Wikipedia pages with the highest (Carpal Tunnel Syndrome and Urinary Incontinence) and lowest (Telerehabilitation and Fall Prevention) page views.

## Discussion

### Principal Findings

The objective of the Editathons within the PT program at Touro College was to disseminate best-evidence PT-relevant health information to the public using principles of health literacy. These principles include assuring that health information for the public is presented in an easy-to-understand format. Based on the persistence of student contributions and total page views, this initiative was successful in accomplishing that goal. Although the doctoral PT student editors had never done this before and may have anticipated editorial criticism or deletions [4], most student contributions persisted. This persistence demonstrates that the Editathon is an effective method for improving the quality of Wikipedia’s health pages. It is important to emphasize that high-quality, timely information is presented in a format that is accessible to typical Wikipedia users. Wikipedia’s history logs show that the Wikipedia community responded to project contributions in all 24 cases and usually within 1 day of the submission. In all cases, the edits enhanced the student contributions and added more information for the public to access. Examining the editorial response to these 24 health pages for three Editathons may not be sufficient to generalize how all reviewing happens in Wikipedia. However, these findings demonstrate that Wikipedia’s internal communication systems facilitate the editorial process by matching reviewers to incoming edits and providing guidelines to assist reviewers in the editorial process [8,9].

An added benefit of the student edits is that these changes evoked interest in the health page by the editorial community. This in turn led to additional edits and inclusion of citations that further enhanced the content quality, improving the delivery of timely information to the reader. This is evident in Figure 2, where it is noted that 23 additional citations were added between February 5, 2018 (the date of the Editathon), and May 31, 2018 (the end of the observation period).

As deficiencies in the translation of research into practice are widely recognized [10-12], the Wikipedia model is one way to close this gap, bringing current, relevant information to the masses. Taking on the role of Wikipedia editor compelled students to gather and synthesize current, best-evidence information, promoting learning [13]. Living reviews, where current research findings are added to existing best-evidence documents, have the potential to expedite the dissemination of current research findings and translation of knowledge to practice. Ultimately, the public benefits from these collective efforts. Despite this systematic approach, there are those who continue to reject Wikipedia as a useful health resource. While one may argue that editors cannot capture all misinformation, and organizers are aware of this limitation [14], Wikipedia remains one of the most accessed health information sites [1].

### Limitations

There are several limitations to this study that must be considered. One limitation is the fact that we chose the same end date for tracking page views, and thus the length of follow-up time for each Editathon varied. Edits that existed longer than others could potentially have had more views. However, to address this limitation we also tracked the number of page views during a 4-month period following each Editathon for a more uniform comparison. As our goal was to report on the reach/dissemination of the added information, the final page view count is relevant. In addition, since there was no established methodology for monitoring the persistence of edits, we devised a systematic approach that has not yet been reproduced. Finally, it is not possible to ascertain if the viewers read the part of the page with the added health information or if the information was useful to the reader. Our data only tells us that the page was viewed.

### Conclusion

Physical therapists and most health care professionals value the importance of patient education for optimizing outcomes. Patient education is key to promoting self-management, knowing when and where to seek care and which treatments are helpful or harmful, and facilitating compliance with lifestyle and medical regimens that promote optimal health. Through three Editathons, 98 students added best-evidence information to 24 Wikipedia pages that were viewed nearly 8 million times with an average of approximately 350,000 views per page. The Wikipedia Editathon is an excellent way of bridging health technology with best-evidence approaches to care and bringing accurate, useful information to the public. Understanding the editing infrastructure built into the Wikipedia framework allows clinicians to effectively use this vast resource. The Editathon is inexpensive and has far-reaching and lasting impact. Future efforts may include interprofessional collaboration and assessment of the utility of health information in Wikipedia target articles.

## Appendix

Multimedia Appendix 1 Wikipedia article page views.

## Abbreviations

PT: physical therapy
PMID: PubMed identifier
